# Assessment of the Correlation and Diagnostic Accuracy between CSF and Plasma AD Biomarkers: A Comparison of the Lumipulse and Simoa Platforms

**DOI:** 10.1002/alz.086616

**Published:** 2025-01-09

**Authors:** Gerard Piñol‐Ripoll, Faride Dakterzada, Raffaela Cipriani, Ricard López Ortega, Alfonso Arias, Iolanda Riba Llena, Maria Ruiz Julián, Raquel P Huerto, Nuria Tahan, Carlos Matute, Estibaliz Capetillo‐Zarate

**Affiliations:** ^1^ Hospital Universitari Santa Maria de Lleida, IRBLleida, Lleida Spain; ^2^ Achucarro Basque Center for Neuroscience, Leioa, Vasc County Spain; ^3^ Hospital Arnau Vilanova de Lleida, LLeida Spain; ^4^ Hospital Universitari Santa Maria, LLeida Spain; ^5^ Hospital Universitari Santa Maria Lleida, Cognitive Impairment Unit, Lleida, Spain, Lleida Spain; ^6^ Achucarro Basque Center for Neuroscience, Leioa Spain; ^7^ University of the Basque Country UPV/EHU, Leioa Spain

## Abstract

**Background:**

Alzheimer’s disease (AD) plasma biomarkers related to amyloid (A), tau (T), and neurodegeneration (N) can potentially be used to identify these pathological features of the disease, as shown in recent studies. Our objective was to compare the clinical and analytical performance of plasma AD biomarkers measured using the single‐molecule array (Simoa) and Lumipulse platforms.

**Method:**

We quantified ATN and AT plasma biomarkers in 127 patients with mild cognitive impairment (MCI) (n = 81), AD (n = 30), and non‐AD dementia (n = 16) using a Simoa HD‐1/HD‐X analyser (Quanterix) and a Lumipulse G600II automated platform (Fujirebio Europe NV).

**Result:**

We found a strong correlation between the Simoa and Lumipulse methods, although there were systematic differences between biomarker values measured by each method. Concerning the clinical diagnosis, Simoa Ptau181/Aβ42 (AUC 0.739, 95% CI 0.592–0.887) and Lumipulse Aβ42 and Ptau181/Aβ42 (AUC 0.735, 95% CI 0.589–0.882 and AUC 0.733, 95% CI 0.567‐0.900) had the highest discriminating power. However, their power was significantly lower than that of CSF Aβ42/Aβ40, as measured by Lumipulse (AUC 0.879, 95% CI 0.766–0.992). Similarly, Simoa Ptau181 and Lumipulse Ptau181/Aβ42 were the markers most consistent with the CSF Aβ42/Aβ40 status (AUC 0.801, 95% CI 0.712–0.890 vs. AUC 0.870, 95% CI 0.806–0.934, respectively) at the ≥2.127 and ≥0.084 cut‐offs, respectively.

**Conclusion:**

The Simoa and Lumipulse plasma AD assays showed comparable clinical and analytical performance. However, the performance of these biomarkers is weaker than that of CSF AD biomarkers. Interestingly, both platforms identify plasma Ptau181/Aβ42 as a promising biomarker for AD. At present, the analysed AD plasma biomarkers may be useful for screening to reduce the number of lumbar punctures in the clinical setting. However, the use of these markers as diagnostic tools requires further investigation.

